# Algorithm for shortest path search in Geographic Information Systems by using reduced graphs

**DOI:** 10.1186/2193-1801-2-291

**Published:** 2013-07-01

**Authors:** Rafael Rodríguez-Puente, Manuel S Lazo-Cortés

**Affiliations:** Universidad de las Ciencias Informáticas, Habana, Cuba

**Keywords:** Shortest path search algorithm; Geographic Information Systems; Network analysis; Reduced graphs; Dijkstra’s algorithm

## Abstract

The use of Geographic Information Systems has increased considerably since the eighties and nineties. As one of their most demanding applications we can mention shortest paths search. Several studies about shortest path search show the feasibility of using graphs for this purpose. Dijkstra’s algorithm is one of the classic shortest path search algorithms. This algorithm is not well suited for shortest path search in large graphs. This is the reason why various modifications to Dijkstra’s algorithm have been proposed by several authors using heuristics to reduce the run time of shortest path search. One of the most used heuristic algorithms is the A* algorithm, the main goal is to reduce the run time by reducing the search space. This article proposes a modification of Dijkstra’s shortest path search algorithm in reduced graphs. It shows that the cost of the path found in this work, is equal to the cost of the path found using Dijkstra’s algorithm in the original graph. The results of finding the shortest path, applying the proposed algorithm, Dijkstra’s algorithm and A* algorithm, are compared. This comparison shows that, by applying the approach proposed, it is possible to obtain the optimal path in a similar or even in less time than when using heuristic algorithms.

## Introduction

From a practical point of view, a Geographic Information System (GIS) is a computer system capable of handling georeferenced data. These kinds of data refer to information associated with geographic coordinates (longitude, latitude). A GIS should also facilitate the relationship between socio-economic data (i.e. population density) and geographic data, this can be achieved through the generation of thematic maps (Jiang et al. 2010), a service for generating this kind of maps is described by (Rodríguez-Torres and Rodríguez-Puente 2010). The relevance of a GIS is closely related to the ability of building models or representations coming from the real world. This kind of system is very important because it facilitates the decision-making process and has a high social impact. Among the most demanded features in GIS we can mention those related to the analysis of routes, some examples are as follows:

- What is the shortest path between places *x* and *y*?
- What is the optimal path between places *x* and *y* considering a certain criterion?
- What is the lowest cost path between *x* and *y* via places *x*_1_, *x*_2_, …, *x*_*n*_?

Shortest path search has been widely studied. Many applications can be found in various branches of science, specifically in GIS. The road networks used by GIS to respond to the above requests are usually large and could have thousands of streets, that is why one should pay particular attention to how such information is processed.

One of the classic and most used algorithms for calculating the shortest path from an origin to a destination is Dijkstra’s algorithm, it was first enunciated by Edsger Wybe Dijkstra (1959) and is one of the most used and discussed algorithms in the literature of graphs, the temporal complexity is *O*(|*E*| + |*V*|*log*|*V*|), where |*E*| is the number of edges and |*V*| is the number of vertices of the graph. However, this algorithm is not efficient for searching shortest path in large graphs (Fuhao and Jiping 2009).

Various modifications to Dijkstra’s algorithm have been proposed by several authors. Some of these algorithms use heuristics to reduce the run time of shortest path search and we can classify them as follows:

1. Without data preprocessing, i.e.:
  A* (A-star) algorithm (Hart et al. 1968). Improved Live long planing A* (Huang et al. 2007).
  Bidirectional search (Pohl 1969).
  In (Nazari et al. 2008) an approach based on restrictions on the search space is proposed.
2. With data preprocessing, i.e.:
  Reach-Based Pruning (Gutman 2004).
  Landmark-A* (Goldberg and Harrelson 2005; Goldberg and Werneck 2005).
  Highway Hierarchies (Geisberger et al. 2008; Jagadeesh and Srikanthan 2008; Sanders and Schultes 2005; Song and Wang 2011; Wang et al. 2006).
  Edge flags (Koehler et al. 2005; Möhring et al. 2006).
  Geometric containers (Wagner and Willhalm 2007).
  Precomputed Cluster Distances (PCD) (Maue et al. 2010).

Delling et al. (2009) show an overview of routing algorithms; all approaches show important advances in shortest path search and make possible a low response time in large graphs using heuristics.

One of the most used heuristic algorithms is the A* algorithm, the main goal is to reduce the run time by reducing the search space analyzing only the vertices that have better possibilities to appear in the shortest path. The results obtained by this algorithm depend on the heuristic function used to determine the order in which vertices are visited. If the selected heuristic is optimal the computational complexity is reduced to *O*(*n*). That is why the A* algorithm is widely used for shortest path search.

One approach studied for shortest path search on large graphs is related to the use of some properties of the road networks, mainly to reduce the search space of the shortest path.

In the following paragraphs we will be referring to some relevant researches:

- Gutman proposes an approach (Gutman 2004) in which he defines a formal attribute of vertex called *reach*, in order to measure vertex relevance. The reach attribute is precalculated using the graph to reduce the run time of shortest path search.
- A relevant approach that uses a property of a road network is related to the hierarchy present in this kind of network. Many strategies use this approach, for example, Sanders and Schultes propose algorithms for constructing and querying highway hierarchies achieving a small run time and show the feasibility of this approach (Sanders and Schultes 2005).
- Bast et al. define an approach based on relevant nodes (transit nodes) for long-distance travel (Bast et al. 2007). It consists of making precalculations of shortest path between all pairs of transit nodes and from each potential source or destination to its access transit nodes. This approach needs an effective notion of “far away” and the optimal results are guaranteed depending on the local filter selected.
- Gonzalez et al. use the hierarchy of roads for partitioning the network into areas and make precalculations of shortest path in these areas (Gonzalez et al. 2007). This approach uses the fact that some roads are more traveled than others and drivers usually use the largest roads.
- Geisberger et al. propose an approach that uses only edges that are related with “important” nodes (Geisberger et al. 2008). Pfoser et al. present a shortest path algorithm that imitates human driving behavior by exploiting road network hierarchies (Pfoser et al. 2009).

As an important characteristic of the approaches described above, it may be determined that they are based on the idea that for calculation of large paths (in large networks), only high levels roads (highways, roads more traversed, etc.) of the hierarchical road network are needed. This consideration can reduce the run time of shortest path search algorithms, but can not guarantee to return the optimal path.

Various commercial systems use heuristic algorithms with the aim of reducing the run time (Bast et al. 2007). Various authors have defined heuristics for achieving this goal (Fei et al. 2010; Liu and Yang 2009; Nazari et al. 2008; Sun et al. 2008; Xu 2005). Fu et al. show a review of this kind of algorithms for shortest path search in transportation applications (Fu et al. 2006).

Heuristic algorithms are relevant for shortest path search in large graphs, even when an error is introduced, acceptable in most of the situations, but they do not guarantee to obtain the optimal path in all cases.

On the other hand, there are algorithms for reducing a graph (Liu et al. 2010; Lu and Liu 2007; Sadiq and Orlowska 2000). With the application of any algorithm on the reduced graph, obviously, a lower response time is achieved. However, in this case, reduction of data brings loss of information. Thus, obtaining a path that is the optimal in the original graph can not be guaranteed.

Rodríguez-Puente proposes a graph reduction algorithm without loss of information (Rodríguez-Puente 2010). It specifies a mechanism to obtain the original graph from which the reduced graph was obtained. This algorithm can be applied naturally to a GIS because a map is usually divided into: zip code, states, regions, etc. This fragmentation of the map contribute to create a partition according to the algorithm requirements. This algorithm has a computational complexity *O*(*n*^4^), which is a high cost for a response in real-time environment. However, in the proposed approach we make a graph reduction for each graph, only once, and the execution of the reduction algorithm is done only for data preprocessing. Highlighting that it does not affect the run time of shortest path search.

This article presents a modification of Dijkstra’s shortest path search algorithm. It shows that it is possible to obtain the lowest cost path in all cases in a time similar to A* algorithm. Thus, the application of this algorithm in GIS can make improvements in services provided by this kind of systems. The use of the proposed algorithm integrated with the mentioned reduction algorithm will ensure efficiency in shortest path search, while maintaining accuracy.

The paper is organized as follows: first, a brief description of the graph reduction algorithm is provided. Second, the algorithm for finding shortest paths in reduced graphs is presented. Then, correctness of the algorithm is proved. Finally, some experimental results and conclusions are discussed.

## Graph reduction

In order to achieve a better understanding of the proposal, certain definitions and notations related with graph theory must be introduced. Then, the selected graph reduction algorithm, used in the proposed approach, is presented.

### Definitions and notations

Relevant definitions and notations related to the proposed approach are as follows:

#### Definition 1

A graph is a pair *G* = (*V*, *E*), where:

- *V* is a set of vertices.
- *E* is a set of edges. An edge is an unordered pair of vertices (*v*_*i*_, *v*_*j*_) such that *v*_*i*_, *v*_*j*_ ∈ *V*.

#### Definition 2

A weighted graph is defined as a structure *G* = (*V*, *E*, *f*_*c*_), where:

- *V* is a set of vertices.
- *E* is a set of edges.
- The function  assigns to each edge a positive real value called cost.

#### Definition 3

A graph rewrite rule *R* = (*G*_*i*_, *G*_*j*_, *ψ*_*in*_, *ψ*_*out*_) over a graph *G* = (*V*, *E*,*f*_*c*_) consists of:

- a graph *G*_*i*_ = ({*v*_*i*_}, *ϕ*), where *v*_*i*_ ∈ *V*.
- a graph *G*_*j*_ = (*V*_*j*_, *E*_*j*_).
- two sets of embedding information *ψ*_*in*_, *ψ*_*out*_ of the form {(*v*_*m*_, *c*_1_, *c*_2_, *v*_*n*_)}, where: ; in the case of *ψ*_*in*_, ∃(*v*_*n*_, *v*_*i*_) ∈ *E*, such that *f*_*c*_(*v*_*n*_, *v*_*i*_) = *c*_1_. After applying the rewrite rule, a new graph *H* = (*V*_1_, *E*_1_, *f*_*c*1_) is obtained and it holds that ∃(*v*_*n*_, *v*_*m*_) ∈ *E*_1_, such that *f*_*c*1_(*v*_*n*_, *v*_*m*_) = *c*_2_. Analogously to *ψ*_*in*_, we define *ψ*_*out*_, with edges orientation as the only difference.
- *V*_1_ = {*V* − {*v*_*i*_}} ∪ *V*_*j*_. *E*_1_ = *E* − *E*_*t*_ ∪ *E*_*j*_ ∪ *E*_*k*_, (*v*_*t*1_,*v*_*t*2_) ∈ *E*_*t*_ if and only if (*v*_*t*1_ = *v*_*i*_ and *v*_*t*2_ ∈ *V*) or (*V*_*t*1_ ∈ *V* and *v*_*t*2_ = *v*_*i*_). (*v*_*m*_, *v*_*n*_) ∈ *E*_*k*_ if and only if (*v*_*m*_, *c*_1_, *c*_2_) ∈ (*ψ*_*in*_ ∪ *ψ*_*out*_), . ,

A graph rewrite rule also can be defined over an undirected graph, in this case, the sets *ψ*_*in*_ and *ψ*_*out*_ must be represented as an only set called *ψ*.

The set of edges that join vertex *v*_*i*_ with the vertices of the graph *G* − *G*_*i*_ are called pre-embedding edges. After applying a rewrite rule, the edges that join a vertex of the graph *G*_*j*_ with a vertex of the graph *G* − *G*_*j*_ are called post-embedding edges. The function *ψ*_*in*_ transforms the set of pre-embedding edges that are incident in a vertex *v*_*i*_ in post-embedding edges that are incident in one or more vertices *v*_*j*_ ∈ *V*_*j*_. Similarly, the function *ψ*_*out*_ transforms pre-embedding outgoing edges from a vertex *v*_*i*_ in one or more post-embedding outgoing edges from several vertices *v*_*j*_ ∈ *V*_*j*_.

#### Definition 4

A reduced graph is a tuple *G*_*r*_ = (*V*_*r*_, *E*_*r*_, *f*, *R*), where:

- *V*_*r*_ is a set of vertices.
- *E*_*r*_ is a set of edges.
, is a function that for each (*v*_*i*_, *v*_*j*_, *v*_*k*_) returns the cost of going from *v*_*i*_ to *v*_*k*_ through *v*_*j*_, with *v*_*k*_ adjacent to *v*_*j*_ and *v*_*j*_ adjacent to *v*_*i*_. Function *f* is obviously also defined for the cases where *v*_*i*_ = *v*_*j*_ and/or *v*_*j*_ = *v*_*k*_. In the trivial case, *f*(*v*, *v*, *v*) = 0.
- *R* is a set of rewrite rules over (*V*_*r*_, *E*_*r*_, *f*_*c*_), where *f*_*c*_ is defined as *f*_*c*_(*v*, *w*) = *f*(*v*, *v*, *w*).

This definition is particularly important when it is associated with another graph, i.e., when a graph is reduced from another graph. We can state that a graph *G*_*r*_ = (*V*_*r*_, *E*_*r*_, *f*, *R*) is reduced from a graph *G* = (*V*, *E*, *f*_*c*_), when applying the set of rewrite rules *R* to the graph *G*_*r*_, the graph *G* is obtained.

In the case of function *f*, for all 3-tuple of vertices *v*_*i*_, *v*_*j*_, *v*_*k*_ ∈ *V*_*r*_ it holds that *f*(*v*_*i*_, *v*_*j*_, *v*_*k*_) = *f*_*c*_(*v*_*i*_, *v*_*j*_) + *f*_*c*_(*v*_*j*_, *v*_*k*_). Notice that *f*(*v*_*i*_, *v*_*i*_, *v*_*j*_) = *f*_*c*_(*v*_*i*_, *v*_*j*_). If *v*_*i*_ and *v*_*j*_ are not adjacent, the image of both functions would be infinite. This is the way in which we specify that two vertices are not adjacent.

### Graph reduction algorithm

The reduction algorithm enunciated in (Rodríguez-Puente 2010) has as a key characteristic that it guarantees no loss of information through the incorporation of rewrite rules. However, an improved version is presented here, since it is necessary to differentiate between what are defined as internal and external vertices below.

This algorithm has two variables as input: a reduced graph *G* = (*V*, *E*, *f*, *R*) and a partition over the set of vertices of the graph. On the other hand, the algorithm has as output, a reduced graph.

In first place, it is necessary to refine partition *P* in order to achieve an optimal path having the same cost of the optimal path obtained by Dijkstra’s algorithm in the original graph; to do this, we introduce the following definition:

#### Definition 5

Let a graph *G* = (*V*, *E*) and a partition *P* on *V*, a vertex *v*_*i*_ ∈ *V* is internal if ∀*v*_*j*_ ∈ *V*, such that *v*_*i*_ and *v*_*j*_ are adjacent, it holds that *v*_*i*_ and *v*_*j*_ are in the same class of *P*; i.e. [*v*_*i*_] = [*v*_*j*_] otherwise *v*_*i*_ is external.

For refining *P*, we use the following strategy:

- Two vertices *v*_*i*_ and *v*_*j*_ are in the same class of refined partition if, and only if:
  - *v*_*i*_ and *v*_*j*_ are in the same class in the original partition *P*.
  - *v*_*i*_ and *v*_*j*_ are internal vertices.
- For each external vertex a new equivalence class is created as a singleton containing only this vertex.

**Figure 1:**
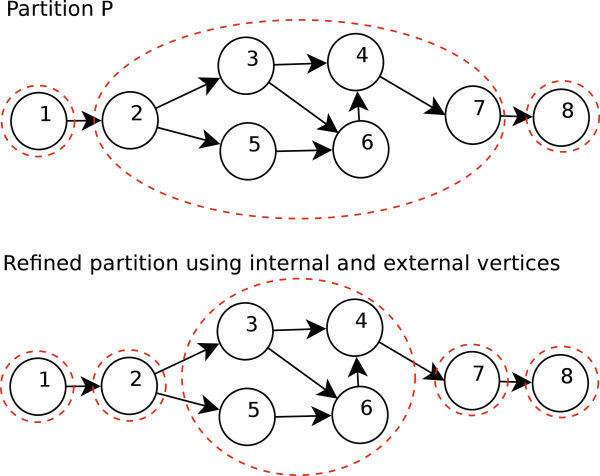
**Example of partition refinement.**

In Figure 1, we show an example of how to refine a partition using definitions of internal and external vertex.

Next, we create a new vertex *w*_*i*_ for each *A*_*i*_∈*P*,|*A*_*i*_|>1,*i*=1..*s*. *V*^′^=*w*_*i*_ is a set of reduced vertices and *V* − *V*^′^ is the set of unreduced vertices in the reduced graph.

We add a vertex in the reduced graph for each class of the partition calculated in the previous step. If the cardinality of the class is 1, the vertex is considered as an unreduced vertex; in any other case, it is considered as a reduced one (*GetReducedVertices* method). Next, a set of edges is calculated. One edge can be added to the reduced graph if the two vertices of the edge belong to different equivalence classes (*GetEdges* method). With the addition of edges to the reduced graph, the cost function *f*_*r*_ of the reduced graph must be updated.

The creation of the set of rewrite rules is an essential step in the reduction algorithm. With the rewrite rules, the original graph can be obtained from the reduced graph. Therefore, rewrite rules guarantee no loss of information, and so the reduction process is reversible.

According to Definition 3, a graph rewrite rule is a quadruple of the form (*G*_*i*_, *G*_*j*_, *ψ*_*in*_, *ψ*_*out*_). Then, we create a rewrite rule for each reduced vertex in *V*^′^, where:

- *G*_*i*_ = ({*w*_*i*_}, *ϕ*), *w*_*i*_ ∈ *V*^′^.
- *G*_*j*_ = (*A*_*i*_, *E*_*i*_, *f*_*cj*_) is a subgraph of *G* = (*V*, *E*, *f*_*c*_, *R*), where exists an edge (*u*, *v*) ∈ *E*_*i*_ if and only if (*u*, *v*) ∈ *E* and *u*, *v* ∈ *A*_*i*_; in addition *f*_*cj*_(*u*, *v*) = *f*_*c*_(*u*, *v*).
- *ψ*_*in*_ is a set of quadruples of the form (*v*_*m*_, *c*_1_, *c*_2_, *v*_*n*_) such that for *v*_*m*_ ∈ *A*_*i*_ and *v*_*n*_ ∈ (*V* − *A*_*i*_) and (*v*_*n*_, *v*_*m*_) ∈ *E* and (*v*_*n*_, *v*_*i*_) ∈ *E*_*r*_ it holds that *c*_1_ = *f*_*cj*_(*v*_*n*_, *v*_*m*_); and *c*_2_ = *f*_*cj*_(*v*_*n*_, *v*_*i*_).
- *ψ*_*out*_ is a set of quadruples of the form (*v*_*m*_, *c*_1_, *c*_2_, *v*_*n*_) such that for *v*_*m*_ ∈ *A*_*j*_ and *v*_*n*_ ∈ (*V* − *A*_*j*_) and (*v*_*m*_, *v*_*n*_) ∈ *E* and (*v*_*i*_, *v*_*n*_) ∈ *E*_*r*_ it holds that *c*_1_ = *f*_*cj*_(*v*_*m*_, *v*_*n*_); and *c*_2_ = *f*_*cj*_(*v*_*i*_, *v*_*n*_).

The previous explanation corresponds to the implementation of *GetRewriteRules* method.

Another step that contributes to obtain the optimal path is the calculation of function *f*_*r*_. Function *f*_*r*_ stores the cost of the shortest path from one vertex to another, traversing a reduced one.

Function *f*_*r*_ is calculated, initially, (*Updatefr* method) for each reduced vertex. This step is made in this way:

- Create an auxiliary graph. First, this graph is equal to the graph *G*_*j*_ = (*V*_*j*_, *E*_*j*_, *f*_*cj*_) of the rewrite rule. Second, we add to this graph, vertices that are adjacent (in the original graph) to vertices of graph *G*_*j*_ (notice that these vertices are internal taking into account original graph and set *v*_*j*_), and the edges that connect them.
- We apply MDijkstra algorithm (see next section) using all pairs of related vertices, identified in the previous step, as origin and destination vertices.
- The obtained costs and path are stored in *f*_*r*_.

Additionally, for all 3-tuples of vertices *v*_*i*_, *v*_*j*_, *v*_*k*_ ∈ *V*, where *v*_*j*_ is a non-reduced vertex, *f*_*r*_(*v*_*i*_, *v*_*j*_, *v*_*k*_) = *f*(*v*_*i*_, *v*_*j*_, *v*_*k*_).

Path from *v*_*i*_ to *v*_*k*_ is also stored, with the goal of avoiding additional run time, when the shortest path search in a reduced graph is retrieved.

Algorithm 1 provides the detailed pseudo-code of the graph reduction algorithm.

#### Algorithm 1 ***GraphReduction***



The complexity of the reduction algorithm would be determined by steps 6-8. According to the above description of *Updatefr*, this method calculates shortest path from all external vertices (taking into account the original graph) of *v*_*j*_ to all vertices of the auxiliary graph.

In a graph obtained from a network in a map, a vertex represents the intersection of two or more lines and an edge represents the connection between two intersections. That is why, in this kind of graph, there are no edges that intersect among them. Thus, we can assume that graphs representing the modeled network through a map are planar.

Moreover, in a graph with these characteristics, the degree of a vertex is generally equal to 4, except in a few cases. Thus it is assumed, without loss of generalization, that the degree of a graph that represents a network of this type is less than or equal to 10. Let *Δ*(*G*^+^) the degree of *G*, the auxiliary graph has, at most, *a* · *Δ*(*G*^+^) vertices. In *Updatefr* method, MDijkstra algorithm is called for each adjacent vertex to any vertex of *v*_*j*_ (see Shortest path search algorithm section for temporal complexity of this algorithm), so the temporal complexity, in the worst case, is: *O*(*a* · *Δ*(*G*^+^) · *a* · *Δ*(*G*^+^) log(*a* · *Δ*(*G*^+^))) = *O*(*Δ*(*G*^+^)^2^ · *a*^2^· log(*a*) + *log*(*Δ*(*G*^+^)))

The terms involving *Δ*(*G*^+^) are constant, so the temporal complexity is *O*(*a*^2^· log(*a*)).

As a conclusion, the temporal complexity of Algorithm 1 is of polynomial order. The reduction process is made only once, as data preprocessing. This preprocessing task causes an increased in the spatial complexity but, with this approach, we can obtain lower run time in every shortest path computation over the reduced graph.

### Reduction example

In this section we explain a very simple example to show the reduction process.

**Figure 2:**
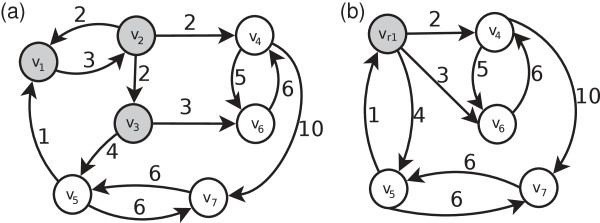
**Two graph examples: (a) is a graph and (b) is a reduced graph from (a).**

Let:

- *G* the graph of Figure 2(a).
- *P* = ({*v*_1_, *v*_2_, *v*_3_}, {*v*_4_}, {*v*_5_}, {*v*_6_}, {*v*_7_}) a partition over the set of vertices of *G*.

In first place, we create the reduced vertices, one per each equivalence class of *P*. Thus, after this step, *G*_*r*_ = ({*v*_*r*1_, *v*_4_, *v*_6_, *v*_5_, *v*_7_}, {}, {}, {}). Notice that *V*_*r*_ = {*v*_*r*1_, *v*_4_, *v*_6_, *v*_5_, *v*_7_}, *E*_*r*_ = {}, *f*_*r*_ = {}, *R*_*r*_ = {}.

Then, we need to calculate the edges of *G*_*r*_ as is specified in the description of Algorithm 1. If there is an edge between two vertices of *G*, and these vertices are unreduced in *G*_*r*_, this edge is added to the reduced graph; for example the edge (*v*_5_, *v*_7_) in *G* is added to *G*_*r*_. Additionally, if there is a vertex *v* ∈ *P*_*i*_ in a class of *P*(*v* ∈ *V*_*r*_), and there exists an edge from *v* to other vertex *u* of *G* (*u* is unreduced vertex in *G*_*r*_), the edge from the reduced vertex, that represents the class *P*_*i*_ of *P*, to the vertex *u* is added to *G*_*r*_; for example the edge (*v*_2_, *v*_4_) in *G* is added to *G*_*r*_ as the edge (*v*_*r*1_,*v*_4_), *v*_2_ is in the class of *P* represented by *v*_*r*1_.

**Figure 3:**
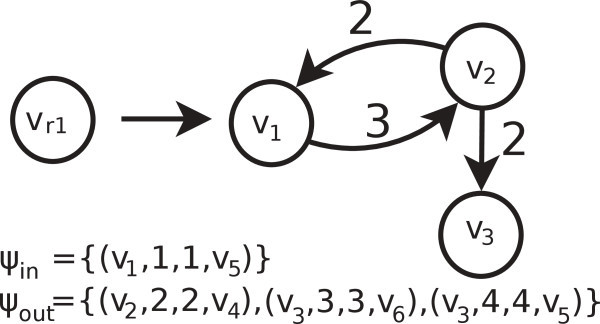
**Rewrite rule example.** On the left side is the graph *G*_*i*_ = ({*v*_*r*1_}, {}), on the right side is the graph *G*_*j*_ = ({*v*_1_, *v*_2_, *v*_3_}, {(*v*_1_, *v*_2_), (*v*_2_, *v*_1_), (*v*_2_, *v*_3_)}) and on the bottom is the embedding information *ψ*_*out*_.

Therefore, the graph of Figure 2(b) is obtained. In addition, the rewrite rules are created. The graph *G*_*i*_ of the rewrite rule is *G*_*i*_ = ({*v*_*r*1_},{}) (see left of Figure 3), the graph *G*_*j*_ is created with the vertices of the class of *P*_*i*_, represented by *v*_*ri*_, and edges among them on *G*, as is presented on the right side of Figure 3. Once we created graphs *G*_*i*_ and *G*_*j*_, the embedding information (*ψ*_*in*_ and *ψ*_*out*_) must be specified, as is described in the specification of the reduction algorithm.

Finally, the function *f*_*r*_ is calculated. In the example of the reduced graph of Figure 2(b), we need to store the path from *v*_5_ to *v*_4_ and the path from *v*_5_ to *v*_6_, both through *v*_*r*1_. In this case, *f*_*r*_(*v*_5_, *v*_*r*1_,*v*_4_) = 6, *f*_*r*_(*v*_5_, *v*_*r*1_,*v*_6_) = 9.

The application of the rewrite rules obtained (Figure 3) to *G*_*r*_ (Figure 2(b)) allows us to obtain the original graph *G* (Figure 2(a)). For this purpose, we enunciated Algorithm 2 based on Definition 3.

This algorithm has as input a reduced graph and a rewrite rule. If a reduced graph has more than one reduced vertex, the application of this algorithm for each reduced vertex would be sufficient to obtain the original graph.

#### Algorithm 2 **Graph Rewrite Rule Application**



Following, we show an example of application of the rewrite rule of Figure 3, using Algorithm 2:

- Add to *G*_*r*_ (Figure 2(b)) the graph *G*_*j*_ of the rewrite rule (*G*_*j*_ is the right side graph of the rewrite rule).
- The pre-embedding edge (*v*_*r*1_, *v*_5_) of cost 1 is transformed in post-embedding edge (*v*_1_, *v*_5_) of cost 1.
- The pre-embedding edge (*v*_*r*1_, *v*_4_) of cost 2 is transformed in post-embedding edge (*v*_2_, *v*_4_) of cost 2.
- The pre-embedding edge (*v*_*r*1_,*v*_6_) of cost 3 is transformed in post-embedding edge (*v*_3_, *v*_6_) of cost 3.
- The pre-embedding edge (*v*_*r*1_,*v*_5_) of cost 1 is transformed in post-embedding edge (*v*_3_, *v*_5_) of cost 4.
- The vertex *v*_*r*1_ is eliminated from *G*_3_.

After applying the rewrite rule we have obtained the graph *G* (Figure 2(a)). Thus, in the reduction process does not exist loss of information, that is, the reduction is reversible.

## Shortest path search algorithm

In this section, a modification of Dijkstra’s shortest path search algorithm is shown. The goal of the proposal is to obtain an optimal path with the same cost as the path returned by Dijkstra’s algorithm, for the same origin and destination, but using a reduced graph.

Both, Dijkstra’s algorithm and the one proposed, are based on iterations over the set of vertices. At each iteration, the algorithm will find a vertex so that the distance from the origin vertex to the selected vertex is minimal. This vertex is called pivot. Usually, the vertices are stored in a priority queue considering, as priority, the distance from the origin vertex. This data structure is used to facilitate the selection of the pivot. Besides, two vectors are updated during the execution of the algorithm. One of them (vector *D*) is updated with the lowest distance from the origin vertex to each vertex *v*_*i*_ (we refer to this distance as *D*[*v*_*i*_]). The other one (vector *P*_*r*_) is updated with the predecessor of each vertex in the shortest path from the origin vertex.

Every time that a pivot *w*_*n*_ is selected, the distances to its adjacent vertices are updated. If the distance from the origin vertex to the pivot (*D*[*w*_*n*_]) plus the distance from the pivot to vertex *v*_*i*_ is lower than the distance from the origin vertex to *v*_*i*_ (*D*[*v*_*i*_]), *D*[*v*_*i*_] is updated.

Additionally, there are two differences between Dijkstra’s algorithm and the proposed one.

In the first place, a cost function *f*:*V* × *V* × *V*→(*R*^+^ ∪ {0, *∞*}) is used for calculating the cost from one unreduced vertex to another one, traversing a reduced vertex. Notice that, traditionally, the cost function of a graph has the cost of an edge.

The other difference in the proposed algorithm, is related to the actualization of distances to a reduced vertex. Let us consider an unreduced vertex *w*_*n*_ as pivot, it is necessary to update the distances to all adjacent vertices as described above. If a reduced vertex *V*_*r*_ is adjacent to the pivot, we have to update the distances to all vertices that are adjacent to *V*_*r*_ (see lines 15-22 of Algorithm 3) using the cost function *f*, for guaranteeing the optimal result.

When analyzing the temporal complexity of the proposed algorithm, there are two differences with respect to Dijkstra’s algorithm. The first one is the use of function *f*, this function is calculated at preprocessing time, so it does not affect the temporal complexity.

The second one implies the execution of one cycle. However, it should be noted that this cycle is repeated *Δ*(*G*^+^) (constant, *Δ*(*G*^+^) < 10) times for each vertex that is stored in the queue.

Thus, *Δ*(*G*^+^) < log(|*V*|) for large graphs, this new cycle does not affect the temporal complexity. Concluding, temporal complexity of Dijkstra and MDijkstra algorithms are the same order. Also notice that, in a planar graph, we can establish a linear relation between vertices and edges. From the Euler’s formula (Diestel 2010), it follows that |*E*| ≤ 3|*V*| − 6 if |*V*| ≥ 3. So, in the case of Dijkstra’s algorithm in planar graphs, we can state that the temporal complexity is *O*(|*E*| + |*V*| log(|*V*|)) = *O*(|*V*| log(|*V*|)).

For applying the proposed approach, we need to reduce a graph only once. Then, we can make several shortest path search computations. In other words, we propose to make a data preprocessing for achieving a performance improvement in shortest path search.

This approach brings us the benefit of performing shortest path search in graphs with less vertices than other algorithms use, for instance, Dijkstra and A*. Therefore, it is logical for the proposal to achieve a lower run time. Nevertheless, it is necessary to demonstrate, that the path obtained by this proposal is optimal and equal (in terms of cost) to the one obtained by Dijkstra’s algorithm. These demonstrations are shown in the following section.

The detailed pseudo-code of the proposed modification is presented in Algorithm 3.

Table 1 shows a comparison of temporal complexity of Dijkstra, A* and MDijsktra algorithms. When analyzing A* algorithm considering optimal heuristics, it can be stated that its temporal complexity is *O*(*n*), where *n* is the number of vertices of the graph. Besides, the temporal complexity of Algorithm 3 (MDijkstra) is *O*(*n*_1_ log(*n*_1_)) < *O*(*n*12), where *n*_1_ is the number of vertices of the reduced graph. Thus, if in the reduction process we obtain a graph *G* = (*V*_*r*_, *E*_*r*_), such that , the temporal complexity of both algorithms must be similar.

**Table 1:** Temporal and spatial complexity of Dijkstra, A* and MDijkstra algorithms

Algorithm	Temporal complexity	Temporal complexity (using Heap data structure)	Spatial complexity
Dijkstra	*O*(|*E*| + |*V*|^2^)	*O*(|*V*| + log(|*V*|))	*O*(|*E*| + |*V*|)
A*	*O*(|*V*|), if the selected heuristic is optimal	*O*(|*V*|), if the selected heuristic is optimal	*O*(|*E*| + |*V*|)
MDijkstra	*O*(|*E*| + |*V*|^2^)	*O*(|*V*| + log(|*V*|))	*O*(|*E*| + |*V*|) + |*R*|

### Algorithm 3 ***mDijkstra***



However, as is impractical to obtain an optimal heuristics for this purpose, we can state that the proposal obtains a response in a lower run time than Dijkstra and A* algorithm if a condition  is satisfied.

Generally, there is a trade off between efficiency and accuracy in algorithms that have large amount of data as input. The main result of the present work is the efficiency improvement of shortest path search in large graphs without affecting accuracy.

We have the possibility to make a shortest path search in the reduced graph between any pair of vertices of the original graph. It can be achieved by applying a rewrite rule to a proper reduced vertex. However, this involves an additional cost to shortest path search.

It is hard to state that an algorithm for shortest path search is better than other in all cases. In this case, our proposal need a higher space, associated to a preprocessing stage to calculate function *f* (see Definition 4), than classical Dijkstra’s and A* algorithms (nevertheless, it should be highlighted that the preprocessing is made only once, but shortest path searches are made several times). However, MDijkstra algorithm gives a response in a lower run time.

Below, we prove the correctness of MDijkstra algorithm, with the aim of establishing that the proposed algorithm obtains an optimal path, and the cost of this path is the same as the cost of the path obtained by Dijkstra’s algorithm. Next, we state a theoretical measure to ensure that the response time is lower than A* algorithm. This is the algorithm selected in the literature of shortest path search, to compare run times.

### Correctness proof

In this paper, a new shortest path search algorithm is proposed. Therefore, it is necessary to prove that the path obtained by the proposal is optimal in all cases.

With the aim of facilitating the understanding of this section, the correctness proof of several lemmas is presented in Appendix A.

By Lemma 3, *D*_*N*−1_(*v*) has the minimum distance from vertex *v*_*o*_ to vertex *v*.

To prove the correctness of Algorithm 3, we shall prove that for any path *Ca* = (*v*_*o*_, *v*_1_, *v*_2_, ..., *v*_*d*_) with distance vector *Dc* and predecessors vector *P*, it holds that ∀*v* ∈ *V*, *D*_*N* − 1_(*v*) ≤ *Dc*_*N*−1_(*v*), where *v* is an unreduced vertex.

#### Theorem 1

∀*n* ∈ {1, 2, .., *N* − 1}[*Ca*(0) = 0 → ∀ *m* < *n* + 1(*Ca*(*m*) < *N*) → ∀*m* < *n* + 1[*Dc*(*Ca*(*m*)) + *f*_*c*_(*Ca*(*m*), *Ca*(*m* + 1)) = *Dc*(*Ca*(*m* + 1))] → *D*_*N*−1_(*Ca*(*n*)) ≤ *Dc*(*Ca*(*n*))]

#### Proof

(By induction on *n*)

Base case *n* = 0 immediate by Lemma 2,

For *n* = *k* + 1:

By Lemma 1 in step *N* − 1 all vertices have been visited. 1

The distance to a vertex *v*_*i*_ is less than or equal to the distance to a visited vertex *v*_*j*_ plus the distance from *v*_*j*_ to *v*_*i*_, by Lemma 5

By induction hypothesis, *D*_*N*−1_(*Ca*(*k* − 1)) ≤ *Dc*(*Ca*(*k* −v1)), replacing *D*_*N*−1_(*Ca*(*k* − 1)) in (1), 2

Note that *Ca*(*k*) = *Pc*_*N*+1_(*Ca*(*k* + 1)) and *Ca*(*k* − 1) = *Pc*_*N*−1_(*Pc*_*N*−1_(*Ca*(*k* + 1))), replacing *Ca*(*k*) y *Ca*(*k* − 1) in (2), 3

By Lemma 3, *Dc*(*Ca*(*k*v+ 1)) = *Dc*(*Pc*_*N*−1_(*Pc*_*N*−1_(*Ca*(*k* + 1)))) + *f*(*Pc*_*N*−1_(*Pc*_*N*−1_(*Ca*(*k* + 1))), *Pc*_*N*−1_(*Ca*(*k* + 1)),*Ca*(*k* + 1)), replacing in (3), *D*_*N*−1_(*Ca*(*k* + 1)) ≤ *Dc*(*Ca*(*k* + 1)). □

We can prove the correctness of Dijkstra’s algorithm with a similar reasoning because the same invariants are satisfied. Thus, for the next proof we assume that Dijkstra’s algorithm is correct and satisfies invariants analogous to those defined for Algorithm 3.

As demonstrated before, Algorithm 3 returns the shortest path in the reduced graph. However, it remains to prove that the cost of the shortest path obtained by the proposed algorithm and the one obtained by Dijkstra’s algorithm (in the original graph without reducing it) are the same.

Let:

- *G* = (*V*, *E*, *f*_*c*_) a graph.
- *G*_*r*_ = (*V*_*r*_, *E*_*r*_, *f*) a reduced graph obtained from the graph *G*.

#### Theorem 2

Let *Ca* = (*v*_1_, ..., *v*_*n*_) be a path of cost *c* obtained by applying Dijkstra’s algorithm on the graph *G*, where *v*_1_ and *v*_*n*_ are unreduced vertices on the graph *G*_*r*_, then ∃*Ca*^′^ = (*u*_1_, *u*_2_, …, *u*_*t*_) with cost *c*, *u*_1_ = *v*_1_, *u*_*t*_ = *v*_*n*_, such that *Ca*^′^ is an optimal path on *G*_*r*_.

#### Proof

From *Ca* we can build a path *Ca*^′^ of cost *c* on the graph *G*_*r*_ as follows:

- Substitute each sub-path *v*_*i*_, *v*_*i*+1_, …, *v*_*i*+*m*_ for a path *v*_*i*_, *v*_*k*_, *v*_*i*+*m*_ where:
  - *v*_*i*+*j*_ ∈ [*v*_*i*_], *j* = 1..*m*
  - *v*_*i*_, *v*_*i*+*m*_ are external vertices. The other vertices are internal
  - *v*_*k*_ is the reduced vertex (in the graph *G*_*r*_) that represents the equivalence class [*v*_*i*_]

The cost of the path *v*_*i*_, *v*_*k*_, *v*_*i*+*m*_ is equal to the cost of the path *v*_*i*_, *v*_*i*+1_, …, *v*_*i*+*m*_, by definition of function *f*. Thus, the paths *Ca* and *Ca*^′^ have the same cost.

Suppose that exists a path *Cb*^′^ = (*u*_1_, *u*_2_, …, *u*_*p*_) of cost *c*_1_ < *c* in the graph *G*_*r*_, where *u*_*i*_ ∈ *V*_*r*_, *i* = 1..*p*. Then we can obtain a path *Cb* of cost *c*_1_ on the graph *G* as follows:

- Substitute each sub-path *u*_*i*−1_,*u*_*i*_, *u*_*i*+1_ by a path *u*_*i*−1_,*u*_*j*_, *u*_*j*+1_, *u*_*j*+*m*_, …, *u*_*i*+1_ of cost *c*_3_ where:
  - *u*_*i*−1_,*u*_*i*+1_ are unreduced vertices
  - *u*_*j*+*t*_ ∈ [*u*_*i*_], *j* = 1..*m*
  - *c*_3_ = *f*(*u*_*i*−1_, *u*_*i*_, *u*_*i*+1_)

Therefore paths *Cb* and *Cb*^′^ have the same cost (*c*_1_), this leads a contradiction. Thus, there is no path that has less cost than *Ca*. □

#### Corollary 1

Let *Ca* = (*v*_1_, ..., *v*_*n*_) a path obtained by applying Dijkstra’s algorithm on graph *G*, ∀*i* ∈ {1, 2, ..., *n*} such that *Ca*[*i*] is an unreduced vertex in *G*_*r*_, it holds that the distance to *Ca*[*i*] is equal to the distance obtained by MDijkstra algorithm on the reduced graph from *v*_1_ to *Ca*[*i*].

Theorem 2 establishes that the cost of the shortest path from a vertex *v*_*i*_ to any vertex *v*_*j*_ (*v*_*i*_ and *v*_*j*_ being unreduced vertices in *G*_*r*_) obtained by applying Algorithm 3 is the same as the cost of the shortest path calculated by Dijkstra’s algorithm in the original graph (without reduction).

The fact that both source and destination must be unreduced vertices could be a limiting factor (in terms of the number of vertices to which one can calculate the shortest path) if one does not have a mechanism that allows obtaining a reduced graph *G*_*ri*_ from *G*_*r*_ where *v*_*i*_ ∈ *V* (*v*_*i*_ is a vertex in the original graph *G* = (*V*, *E*)) is an unreduced vertex on *G*_*ri*_. This can be accomplished by one or more expansions applying rewrite rules to the reduced vertex that contains vertex *v*_*i*_.

### Experimental results

The comparison of the results of shortest path search, applying Algorithm 3 (MDijkstra), Dijkstra’s algorithm and A* algorithm, provides elements emphasizing the advantages of the proposed approach. Besides, correctness proof of the proposed shortest path search algorithm is made.

Algorithm 3 was coded in Python, using the NetworkX library (Hagberg et al. 2008). This library provides an implementation of Dijkstra’s and A* algorithms, allowing to compare the three algorithms on the same technology and with efficient data structures. NetworkX uses a priority queue, implemented with a Heap, to find the shortest path using Dijkstra and A* algorithms. With this implementation, the complexity is *O*(|*E*| + |*V*| log(|*V*|)).

It is well-known that there are several techniques to make performance improvement on shortest path search, based on Dijkstra’s and A* algorithms; Zeng and Church compare some of them (Zeng and Church 2009). This performance improvement depends on several things, for example: programming language, data structures used in the implementation of algorithms, among others. Therefore, in order to be impartial with the proposal, we compare the proposed algorithm only with the implementation of Dijkstra’s and A* algorithms in the NetworkX library.

The algorithms were run on a Pentium 4 (3.2 GHz) with 1.5 Gb of RAM and the Kubuntu 11.10 operating system.

Two graphs were used for experimental test: one was obtained from a cartography of the North Carolina State^a^ and the other represents the road network of San Francisco^b^. The first graph, obtained from North Carolina cartography, has 41810 vertices. This graph was reduced twice. First, we arbitrarily construct two sets of polygons using zip codes. The first one has 30 polygons. The second one has 5 polygons (the second set of polygons does not depend of the first one). Obviously in the second case polygons are larger. In both reductions we use the equivalence relation “in”. If two points are into the same polygon, then they are related through relation “in”. We obtain a reduced graph of 1826 using the first set of polygons, and a reduced graph of 250 vertices using the second set.

The second graph, obtained from San Francisco cartography, has 149756 vertices and it was also reduced twice, using the equivalence relation defined above and two new arbitrary sets of polygons. The first set has 10 polygons and the second one has 4 polygons. In the first reduction, using the first set of polygons we obtain a reduced graph of 2617 vertices. Using the second set of polygons, we obtain another reduced graph of 769 vertices.

Dijkstra’s and A* algorithms were executed on the original graphs and the proposed algorithm was applied to the reduced ones. Each algorithm was executed 10 times; the highest and lowest values were discarded. Finally, the average time among the remaining 8 values are shown.

Table 2 shows a comparison among the three selected algorithms based on the run time of shortest path search.

**Table 2:** Time of shortest path search with Dijkstra’s and A* algorithms in two original graphs (*G*_**1**_**,*G***_**2**_**) and time of shortest path search in four reduced graphs with the proposed approach**

Graph	Algorithm	NV^a^	Time (seconds)	Optimal path
*G*_1_	Dijkstra	41810	0.6160	yes
	A* (h=0)		0.4938	yes
	A* (h=Euclidean distance)		0.0200	no
*G*_*r*1.1_	Algorithm 3 (MDijkstra)	250	0.0036	yes
*G*_*r*1.2_	Algorithm 3 (MDijkstra)	1826	0.0265	yes
*G*_2_	Dijkstra	149756	3.0249	yes
	A* (h=0)		2.2108	yes
	A* (h=Euclidean distance)		0.1011	no
*G*_*r*2.1_	Algorithm 3 (MDijkstra)	765	0.0193	yes
*G*_*r*2.2_	Algorithm 3 (MDijkstra)	2617	0.0722	yes

^a^Number of vertices of the graph.

## Discussion

The results shown in Table 2 confirm the fact that, for large graphs, the run time of shortest path search with the proposed approach would be smaller than the run time obtained with classical approaches.

If in the reduction process we obtain a graph *G* = (*V*_*r*_, *E*_*r*_), such that , the temporal complexity of both algorithms (Dijkstra and MDijkstra) must be similar. However, as is impractical to obtain an optimal heuristics for this purpose, we can state that the proposal obtains a response in a lower run time than Dijkstra’s and A* algorithm if a condition  is satisfied. Thus, if we assume that we have sufficient memory for storing reduced graphs, the proposed approach is better than Dijkstra’s and A* algorithms; taking into account that if we reduce original graph as proposed before, always we can obtain a response in a lower runtime. The proposal is not useful when the available memory is low and does not permit to store reduced graphs.

In the case of the run time of Algorithm 3 (MDijkstra) on the graph *G*_*r*1.2_, the obtained time is higher than the one obtained by A* algorithm. The reason of this result is that the graph *G*_*r*1.2_ has a number of vertices considerably higher than the square root of the number of vertices of *G*_1_. Notice that we state that the number of vertices of the reduced graph must be less than or equal to the square root of the number of vertices of the original graph. In the case of the graph *G*_*r*2.2_ a lower run time than the one obtained by A* algorithm is achieved, although the number of vertices is higher than the square root of the number of vertices of *G*_2_.

The selection of origin and destination of the shortest path search in a GIS is usually made using a map, i.e. a user selects these points by clicking in the map shown by the GIS. We believe that, at any time that a user selects an origin or a destination point, the GIS can make an expansion of the reduced graph, using the extent of the map that is visualized and the selected point. If a system for shortest path search is implemented in this way, the time needed to expand a reduced vertex would be irrelevant for the shortest path search, considering that the temporal complexity of expanding a reduced vertex is *O*(*a*), where *a*=*max*{|*A*_*i*_|,*A*_*i*_ ∈ *P*}.

Most algorithms developed lately for shortest path search make efficiency improvement by reducing the search space, these approaches cause loss in accuracy. The presented approach makes use of a graph reduction algorithm without loss of information, in order to obtain a better run time of the search. This approach maintains the accuracy because the reduction algorithm guarantees no loss of data (see Table 1).

Generally, heuristic algorithms are developed in order to reduce the run time of a specific algorithm, which solves some problems whose optimal solution involves a high computational cost. Many heuristic algorithms are developed for shortest path search in GIS, with the assumption that a low bound of error is admissible in this area. However, with the proposed approach, it is possible to obtain the optimal path in a similar time, and even in less time, than with heuristic algorithms, as shown in Table 2.

## Conclusions

In this paper, an algorithm for shortest path search on reduced graphs is developed. Experimental results show that the proposed algorithm is more efficient than Dijkstra’s algorithm on large graphs. In addition, we can conclude the following:

- The proposed approach is particularly applicable to GIS, due to the way in which users perform a shortest path search in this kind of systems. This allows us to expand vertices avoiding the influence of the time used in this operation on the shortest path search.
- The use of reduced graphs significantly reduces the response time in the shortest path search. That is one of the two main approaches used in literature to reduce the computational cost of this operation.
- The shortest path search on a reduced graph ensures scalability regarding the size of the graph on which the analysis is performed.
- We prove that the proposed algorithm allows us to obtain an optimal path in a reduced graph. The cost of the obtained path is equal to the cost of the path found using Dijkstra’s algorithm on the original graph.
- We have developed a method capable of performing shortest path search in a run time similar to A* algorithm (with h=0 and h=Euclidean distance).

## Future work

The modifications made on Dijkstra’s algorithm are related to the use of a new function that has the cost of going through a reduced vertex. Therefore, we can modify other algorithms to make shortest path search in reduced graph (like A* algorithm), whenever the cost of going through a reduced vertex is considered as the cost of the path.

## Appendix

### A Demonstration of cycle invariants of Algorithm 3

**Figure 4:**
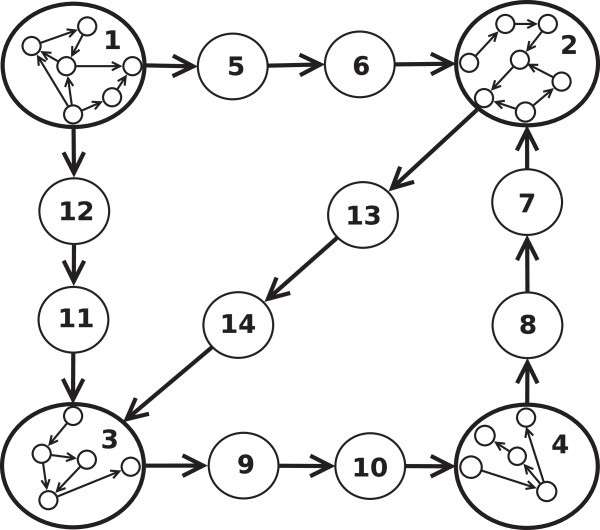
**Reduced graph example.** Vertices 1,2,3 and 4 are reduced vertices, the rest are unreduced ones.

Preconditions that must be met to prove the correctness of Algorithm 3 are expressed by the following definitions and notations:

- *G* = (*V*, *E*, *f*_*c*_) is a weighted graph. Without loss of generality we assume that *V* = {0, 1, ..., *M* − 1} to make demonstrations less complex.
- *G*_*r*_ = (*V*_*r*_, *E*_*r*_, *f*, *R*) is a reduced graph from *G* and the equivalence relation *RE*. Without loss of generality we assume that *V*_*r*_ = {0, 1, ..., *N* − 1}. It is important to notice that in each path of a reduced graph, between two reduced vertices there are, at least, two unreduced vertices, as is shown in Figure 4.
- ∀*n* < *N*, in the execution of Algorithm 3 we define:
  - A vertex *w*_*n*_, the vertex selected in step *n*.
  - A set *C*_*n*_ ⊆ *V*_*r*_, the set of vertices visited in step *n*. *C*_0_ = {*v*_*o*_}, .
  - *D*_*n*_ represents the minimum distance from *v*_*o*_ to each vertex *v* ∈ *V*_*r*_ as far as it is known in step *n*. *D*_0_(*v*_*o*_) = 0, *D*_*n*+1_(*v*) = *Min*(*D*_*n*_(*w*_*n*_) + *f*_*c*_(*w*_*n*_, *v*), *D*_*n*_(*v*)) = *Min*(*D*_*n*_(*P*_*n*_(*w*_*n*_)) + *f*(*P*_*n*_(*w*_*n*_), *w*_*n*_, *v*), *D*_*n*_(*v*)).
  - *P*_*n*_ store, for each vertex, the predecessor in the shortest path from *v*_*o*_ to *v*_*d*_, as far as it is known in step *n*. *P*_0_(*v*_*o*_) = *v*_*o*_,

For the correctness proof it is necessary to demonstrate that the following cycle invariants are held:

∀*n* < *N*:

1. |*C*_*n*_| = *n* + 1. In the iteration *n*, there are *n* + 1 visited vertices.
2. *D*_*n*_(*v*_*o*_) = 0 ∧ *P*_*n*_(*v*_*o*_) = *v*_*o*_. The distance from origin vertex to itself is 0 at any iteration. The predecessor of the origin vertex is the vertex itself.
3. *D*_*n*_(*v*) = *D*_*n*_(*P*_*n*_(*P*_*n*_(*v*))) + *f*(*P*_*n*_(*P*_*n*_(*v*)), *P*_*n*_(*v*), *v*). The distance to a vertex depends on the distance to its predecessor in the shortest path.
4. ∀*v* ∈ *C*_*n*_*D*_*n*+1_(*v*) = *D*_*n*_(*v*). The distance to a vertex in the step *n* is the same that the distance in the step *n* + 1, for all visited vertices.
5. ∀*v*_*i*_, *v*_*j*_ ∈ *V*[*v*_*j*_ ∈ *C*_*n*+1_ → *D*_*n*+1_(*v*_*i*_) ≤ *D*_*n*+1_(*P*_*n*_(*v*_*j*_)) + *f*(*P*_*n*_(*v*_*j*_), *v*_*j*_, *v*_*i*_)]. The distance to any vertex *v*_*i*_ is less than or equal to the distance to a visited vertex *v*_*j*_ plus the distance from *v*_*j*_ to *v*_*i*_.

#### Lemma 1

∀*n* < *N*, |*C*_*n*_| = *n* + 1

#### Proof

(By induction on *n*)

From the definition of the algorithm, at each step a vertex *w* is visited, in step 0 vertex *v*_*o*_ is visited, thus in the base case we have *C*_0_ = {*v*_*o*_}, |*C*_0_| = 1,

For *n* = *k* + 1, , being *v* the visited vertex in step *k* + 1, therefore |*C*_*k*+1_| = |*C*_*k*_| + |{*v*}| = *k* + 2. □

#### Lemma 2

∀*n* < *N*, *D*_*n*_(*v*_*o*_) = 0 ∧ *P*_*n*_(*v*_*o*_) = *v*_*o*_

#### Proof

First, we visit vertex *v*_*o*_ and update *D*_*n*_(*v*_*o*_) = 0, i.e., the minimum distance from *v*_*o*_ to itself is 0, the function *D*_*n*_ has its domain in , so the smallest possible value that can be achieved is 0;

Let *cost* = *D*_*n*_(*P*_*n*_(*w*_*n*_)) + *f*(*P*_*n*_(*w*_*n*_), *w*_*n*_, *v*), ∀*w*_*n*_, *v* ∈ *V*, it holds that 0≤0+*cost*, because the image of the function *f* is  and the vector *D*(*V*_*r*_) is initialized from *f*.

The condition *D*_*n*_(*v*_*o*_) > *D*_*n*_(*w*_*n*_) + *f*(*P*_*n*_(*w*_*n*_), *w*_*n*_, *v*_*o*_) is never satisfied, thus *D*_*n*_[*v*_*o*_] and *P*_*n*_[*v*_*o*_] never change. □

#### Lemma 3

∀*n* < *N*, *D*_*n*_(*v*) = *D*_*n*_(*P*_*n*_(*P*_*n*_(*v*))) + *f*(*P*_*n*_(*P*_*n*_(*v*)), *P*_*n*_(*v*), *v*)

#### Proof

(By induction on *n*)

The base case *n* = 0, ∀*v* ∈ *V*_*r*_, *D*_0_(*v*) = *f*_*c*_(*v*_*o*_, *v*), by preconditions.

*f*(*v*_*o*_, *v*_*o*_, *v*) = *f*_*c*_(*v*_*o*_, *v*_*o*_) + *f*_*c*_(*v*_*o*_, *v*) = *f*_*c*_(*v*_*o*_, *v*), by definition of *f* and *f*_*c*_, replacing *f* by *f*_*c*_:

*D*_0_(*v*) = 0 + *f*(*v*_*o*_, *v*_*o*_, *v*)*D*_0_(*v*) = *D*_0_(*v*_*o*_) + *f*(*v*_*o*_, *v*_*o*_, *v*), by Lemma 2

*D*_0_(*v*) = *D*_0_(*P*_0_(*v*_*o*_)) + *f*(*P*_0_(*v*_*o*_), *v*_*o*_, *v*), by Lemma 2

For *n* = *k* + 1:

Choose *w*_*k*+1_ ∈ *V* ∖ *C*_*k*_ such that *D*_*k*+1_(*w*_*k*+1_) is minimal, .

Case 1: If *D*_*k*+1_(*v*) > *D*_*k*+1_(*P*_*k*+1_(*w*_*k*+1_)) + *f*(*P*_*k*+1_ (*w*_*k*+1_), *w*_*k*+1_, *v*), then *D*_*k*+1_(*v*) = *D*_*k*+1_(*P*_*k*+1_ (*w*_*k*+1_)) + *f*(*P*_*k*+1_(*w*_*k*+1_), *w*_*k*+1_, *v*) ∧ *P*_*k*+1_(*v*) = *w*_*k*+1_

Case 2: If case 1 is not satisfied, *D*_*k*+1_(*v*) = *D*_*k*_(*v*), *P*_*k*+1_(*v*) = *P*_*k*_(*v*), *D*_*k*+1_(*v*) = *D*_*k*_(*P*_*k*_(*P*_*k*_(*v*))) + *f*(*P*_*k*_(*P*_*k*_(*v*)), *P*_*k*_(*v*), *v*), by induction hypothesis, replacing *P*_*k*_(*v*) by *P*_*k*+1_(*v*) *D*_*k*+1_(*v*) = *D*_*k*_(*P*_*k*+1_(*P*_*k*+1_(*v*))) + *f*(*P*_*k*+1_(*P*_*k*+1_(*v*)), *P*_*k*+1_(*v*), *v*). □

#### Lemma 4

∀*v* ∈ *C*_*n*_*D*_*n*+1_(*v*) = *D*_*n*_(*v*)

#### Proof

Let *v* ∈ *C*_*n*_, *w*_*n*_ ∈ *V* ∖ *C*_*n*_

*w*_*n*_ ∈ *C*_*n*+1_ by definition.

*D*_*n*_(*v*) ≤ *D*_*n*_(*w*_*n*_), otherwise vertex *w*_*n*_ was visited before vertex *v*,

*D*_*n*_(*v*) ≤ *D*_*n*_(*w*_*n*_) + *f*_*c*_(*w*_*n*_, *v*) = *D*_*n*_(*P*_*n*_(*w*_*n*_)) + *f*(*P*_*n*_(*w*_*n*_), *w*_*n*_, *v*),

*D*_*n*+1_(*v*) = *D*_*n*_(*v*), by definition of *D*_*n*+1_(*v*). □

#### Lemma 5

∀*n* < *N*, ∀*v*_*i*_, *v*_*j*_ ∈ *V*[*v*_*j*_ ∈ *C*_*n*+1_ → *D*_*n*+1_(*v*_*i*_) ≤ *D*_*n*+1_(*P*_*n*_(*v*_*j*_)) + *f*(*P*_*n*_(*v*_*j*_), *v*_*j*_, *v*_*i*_)]

#### Proof

(By induction on *n*)

The base case *n* = 0, *C*_0_ = {*v*_*j*_}, *D*_0_(*v*_*j*_) = 0, by definition, notice that *v*_*j*_ is the only vertex in *C*_0_ (in the base case, if *v*_*j*_ ∈ *C*_0_, *v*_*j*_ is the origin vertex).

*P*_*n*_(*v*_*j*_) = *v*_*j*_ by Lemma 2.

∀*v*_*i*_ ∈ *V*, *f*(*v*_*j*_, *v*_*j*_, *v*_*i*_) = *f*(*P*_0_(*v*_*j*_), *v*_*j*_, *v*_*i*_), by definition of *f*, and *D*_0_(*v*_*i*_) = *f*(*v*_*j*_, *v*_*j*_, *v*_*i*_). Thus *D*_0_(*v*_*i*_) ≤ 0 + *f*(*P*_0_(*v*_*j*_), *v*_*j*_, *v*_*i*_), *D*_0_(*v*_*j*_) = 0 = *D*_0_(*P*_0_(*V*_*j*_)) *D*_0_(*v*_*i*_) ≤ *D*_0_(*P*_0_(*v*_*j*_)) + *f*(*P*_0_(*v*_*j*_), *v*_*j*_, *v*_*i*_)

*D*_1_(*v*_*i*_) ≤ *D*_0_(*v*_*i*_), from the definition (*D*_*n*+1_(*v*) = *Min*(*D*_*n*_(*w*_*n*_) + *f*_*c*_(*w*_*n*_, *v*), *D*_*n*_(*v*))) and *D*_1_(*v*_*j*_) = *D*_0_(*v*_*j*_) = 0 (notice that *v*_*j*_ is the origin vertex). Replacing *D*_0_ by *D*_1_:

*D*_1_(*v*_*i*_) ≤ *D*_1_(*P*_0_(*v*_*j*_)) + *f*(*P*_0_(*v*_*j*_), *v*_*j*_, *v*_*i*_)

For *n* = *k* + 1:

Case 1: *v*_*j*_ ∈ *C*_*k*_*D*_*k*+1_(*v*_*i*_) ≤ *D*_*k*_(*v*_*i*_), by definition *D*_*k*+1_(*v*_*i*_) ≤ *D*_*k*_(*P*_*k*_(*v*_*j*_)) + *f*(*P*_*k*_(*v*_*j*_), *v*_*j*_, *v*_*i*_), by induction hypothesis *D*_*k*+1_(*v*_*i*_) ≤ *D*_*k*+1_(*P*_*k*_(*v*_*j*_)) + *f*(*P*_*k*_(*v*_*j*_), *v*_*j*_, *v*_*i*_) by Lemma 4

Case 2: *v*_*j*_ = *w*_*k*_.

*D*_*k*+1_(*v*_*i*_) ≤ *D*_*k*_(*P*_*k*_(*w*_*k*_)) + *f*(*P*_*k*_(*w*_*k*_), *w*_*k*_, *v*_*i*_), by definition *D*_*k*+1_(*v*_*i*_) ≤ *D*_*k*+1_(*P*_*k*_(*w*_*k*_)) + *f*(*P*_*k*_(*w*_*k*_), *w*_*k*_, *v*_*i*_), by Lemma 4, replacing *w*_*n*_ by *v*_*j*_:

*D*_*k*+1_(*v*_*i*_) ≤ *D*_*k*+1_(*P*_*k*_(*v*_*j*_)) + *f*(*P*_*k*_(*v*_*j*_), *v*_*j*_, *v*_*i*_). □

## Endnotes

^a^Available in http://grass.osgeo.org/sampledata/north_carolina/

^b^Available in http://www.cs.fsu.edu/~lifeifei/SpatialDataset.htm
